# Supplementary material for: Individual face- and house-related eye movement patterns distinctively activate FFA and PPA
Source: Nat Commun. 2019 Dec 4;10:5532. doi: 10.1038/s41467-019-13541-3 (PMC6892816; doi:10.1038/s41467-019-13541-3)
Supplement: Supplementary file 1 — Supplementary Information [file 41467_2019_13541_MOESM1_ESM.pdf]

Individual face and house-related eye movement patterns distinctively activate FFA and PPA

Lihui Wang[1,2,3,4], Florian Baumgartner[1], Falko R. Kaule[1], Michael Hanke[5,6], and Stefan Pollmann[1,2,7]

[1]Department of Experimental Psychology, Otto-von-Guericke University, Magdeburg, Germany
[2]Center for Behavioral Brain Sciences, Magdeburg, Germany
[3]Institute of Psychology and Behavioral Science, Shanghai Jiao Tong University, Shanghai, China
[4]Shanghai Key Laboratory of Psychotic Disorders, Shanghai Mental Health Center, Shanghai Jiao Tong University School of Medicine, Shanghai, China
[5]Institute of Neuroscience and Medicine, Brain & Behaviour (INM-7), Research Centre Jülich, Jülich, Germany
[6]Institute of Systems Neuroscience, Medical Faculty, Heinrich Heine University Düsseldorf, Düsseldorf, Germany
[7]Beijing Key Laboratory of Learning and Cognition and School of Psychology, Capital Normal University, Beijing, China

Supplementary Table

Supplementary Table 1: Parameters of the Fixation pattern task: Mean Saccadic amplitude, number, fixation duration and dispersion of saccades (mean distance/standard deviation) separated for the two subjects in Experiments 1 and 2 are shown. Here the gaze-tracks were collected from sub11 and sub12 in Experiment 1, while from sub21 and sub22 in Experiment 2 (inv. face: inverted face; controlled: the dispersion of house-fixations was matched to the dispersion of the face-fixations, so they have the same mean distance and standard deviation). For Experiment 3, the same parameters are shown for each participant.

	Saccadic amplitude [°]		Number of saccades		Fixation duration [ms]		Mean dispersion [°]	
**Experiment 1**								
	Face	House	Face	House	Face	House	Face	House
sub11	1.419	1.648	6.67	6.55	396.4	381.4	0.841/1.459	2.228/1.385
sub12	1.390	1.700	6.16	6.81	454.3	430.6	0.794/1.39	2.140/1.287

	Saccadic amplitude [°]			Number of saccades			Fixation duration [ms]			Mean dispersion [°]
**Experiment 2**										
	Face	inv. Face	House	Face	inv. Face	House	Face	inv. Face	House	Controlled
sub21	2.316	2.481	1.940	11.59	11.69	11.88	251.8	242.7	245.9	1.631/1.563
sub22	2.292	1.575	2.032	9.28	10.05	11.52	331.2	305.2	262.7	1.585/1.434

Experiment 3

	Saccadic amplitude [°]		Number of saccades		Fixation duration [ms]		Mean dispersion [°]	
	**Face**	**House**	**Face**	**House**	**Face**	**House**	**Face**	**House**
sub301	2.287	4.336	2.15	2.35	265.5	253.0	1.947/0.574	1.844/1.168
sub302	4.444	6.035	3.03	2.74	193.5	218.5	1.385/0.734	1.693/1.035
sub303	3.163	4.874	2.58	2.98	232.7	195.8	1.429/0.955	1.942/0.947
sub304	4.934	5.047	3.12	2.92	181.9	205.6	1.590/0.970	1.979/1.147
sub305	3.083	4.043	3.12	2.90	191.7	205.8	1.454/0.656	1.446/0.856
sub306	4.283	5.035	2.58	2.38	228.6	248.3	1.512/0.771	1.558/0.975
sub307	3.410	3.653	2.90	2.55	195.4	233.0	1.399/0.978	1.498/1.088
sub308	5.224	4.714	2.69	2.33	217.6	241.7	1.807/0.869	1.451/0.956
sub309	2.870	4.712	2.74	2.76	215.2	211.7	1.754/0.573	1.676/0.888
sub310	3.449	4.561	2.41	2.40	246.4	247.7	1.417/1.096	1.489/1.018
sub311	3.554	5.756	2.54	2.73	218.1	211.7	1.931/0.694	1.960/1.079
sub312	3.021	3.941	2.67	2.62	221.0	232.1	1.352/1.092	1.334/0.826
sub313	4.427	5.675	2.76	2.57	208.4	229.3	1.594/0.776	1.769/1.076
sub314	3.227	5.059	2.41	2.48	242.4	236.4	1.094/0.784	1.610/0.992
sub315	4.680	7.141	2.55	2.35	224.7	244.2	1.831/1.216	2.043/1.166
sub316	3.014	4.391	2.14	2.25	283.8	269.6	1.034/0.456	1.481/0.844

Supplementary Figures

Experiment 1

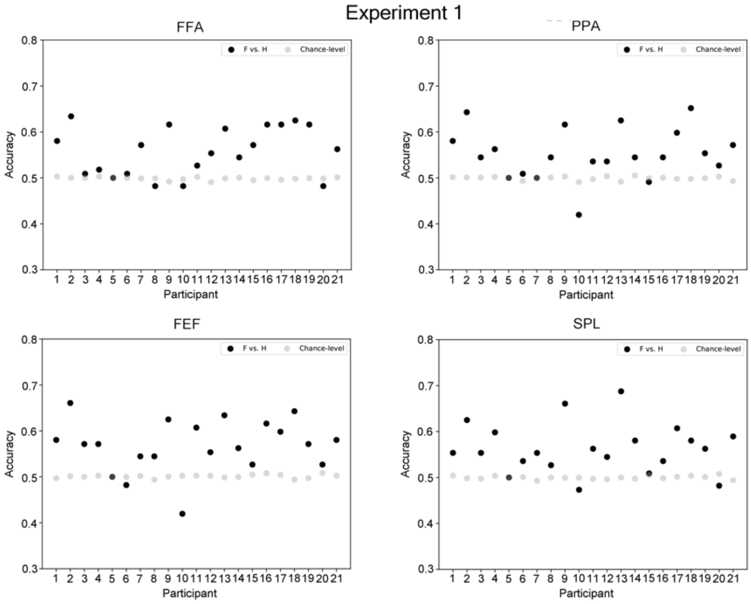

Experiment 2

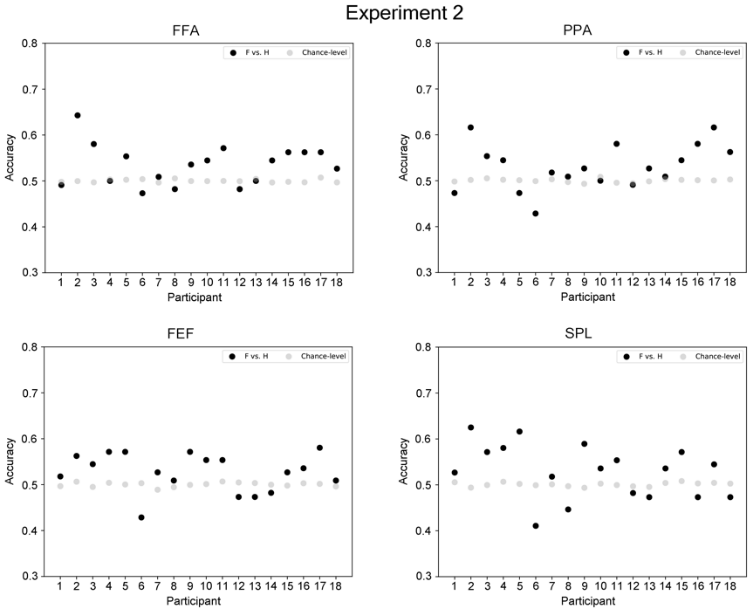

Experiment 3

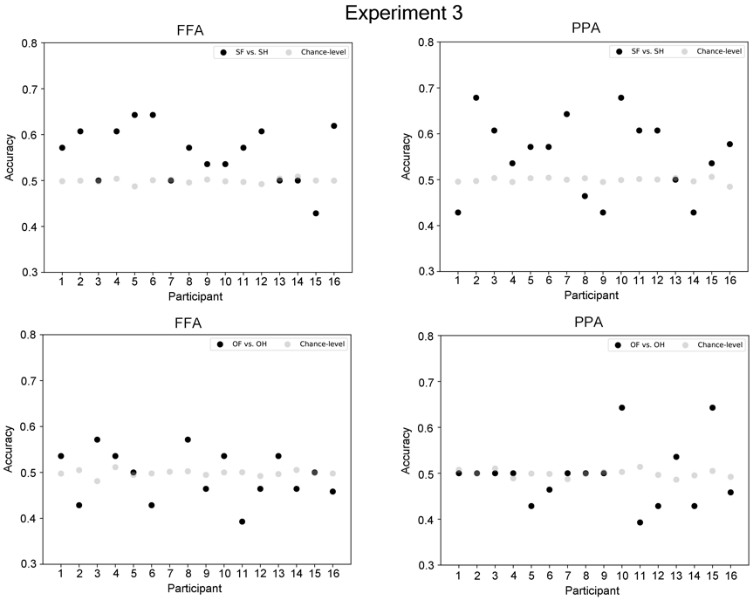

Supplementary Figure 1.

The individual classification accuracies (black dots) and the mean of permutation-based chance accuracies (gray dots) for the Face vs. House (F vs. H) comparison in Experiment 1 and 2 and the Self face vs. Self house (SF vs. SH) and Other face vs. Other house (OF vs. OH) comparisons in Experiment 3.

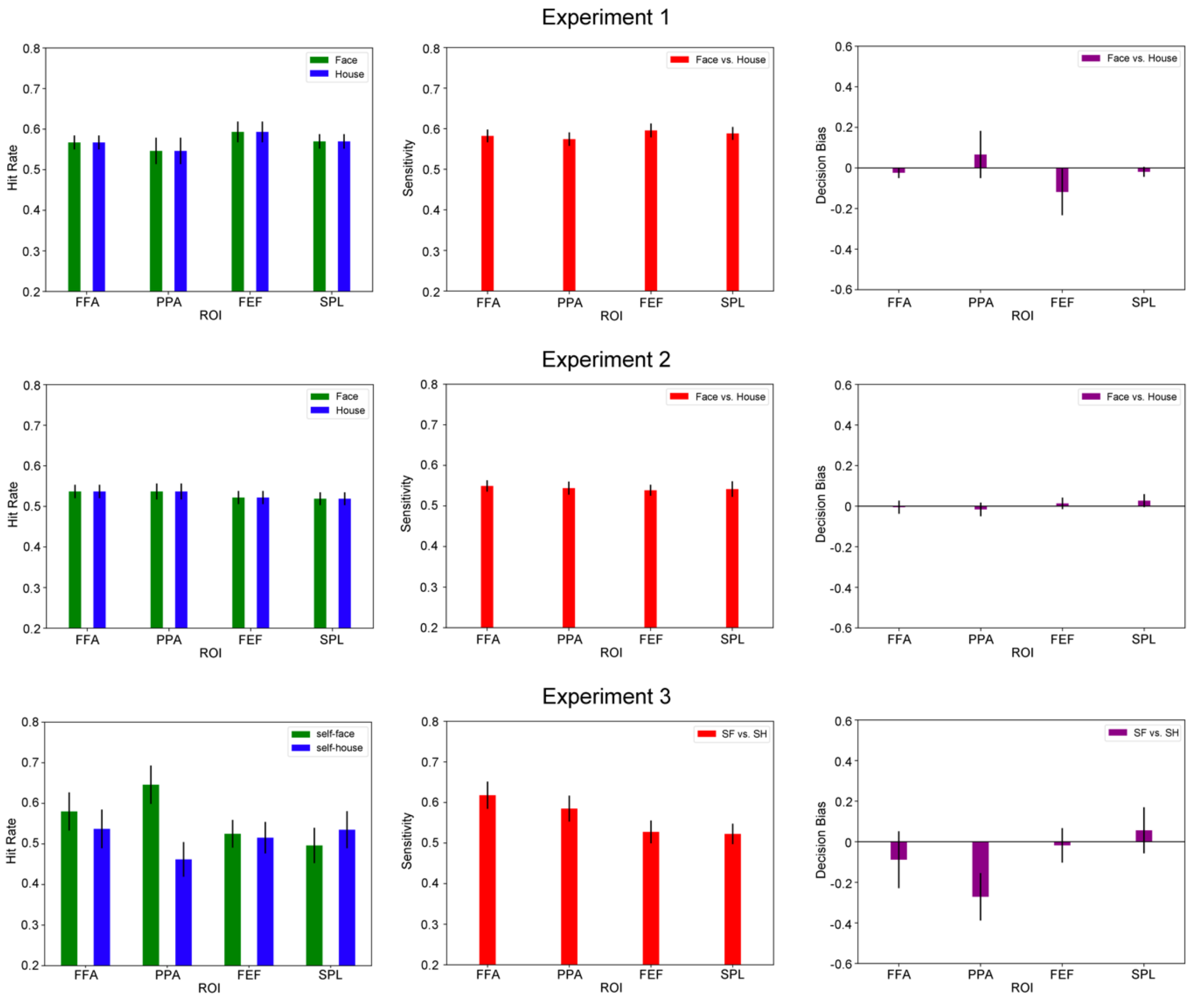

Supplementary Figure 2.

Mean and s.e.m. (shown as error bar) of the hit rate, sensitivity in terms of area under the ROC curve (AUC), and the decision bias in classifying the neural activity patterns associated with the gaze-tracks in the three experiments. A negative value of decision bias indicates a bias for Face (or Self-face in Experiment 3), while a positive value of decision bias indicates a bias for House (or Self-house in Experiment 3). Here to avoid redundancy, the false alarm rates are not illustrated because the false alarm rate of face/self-face equals to 1-hit rate of house/self-house.

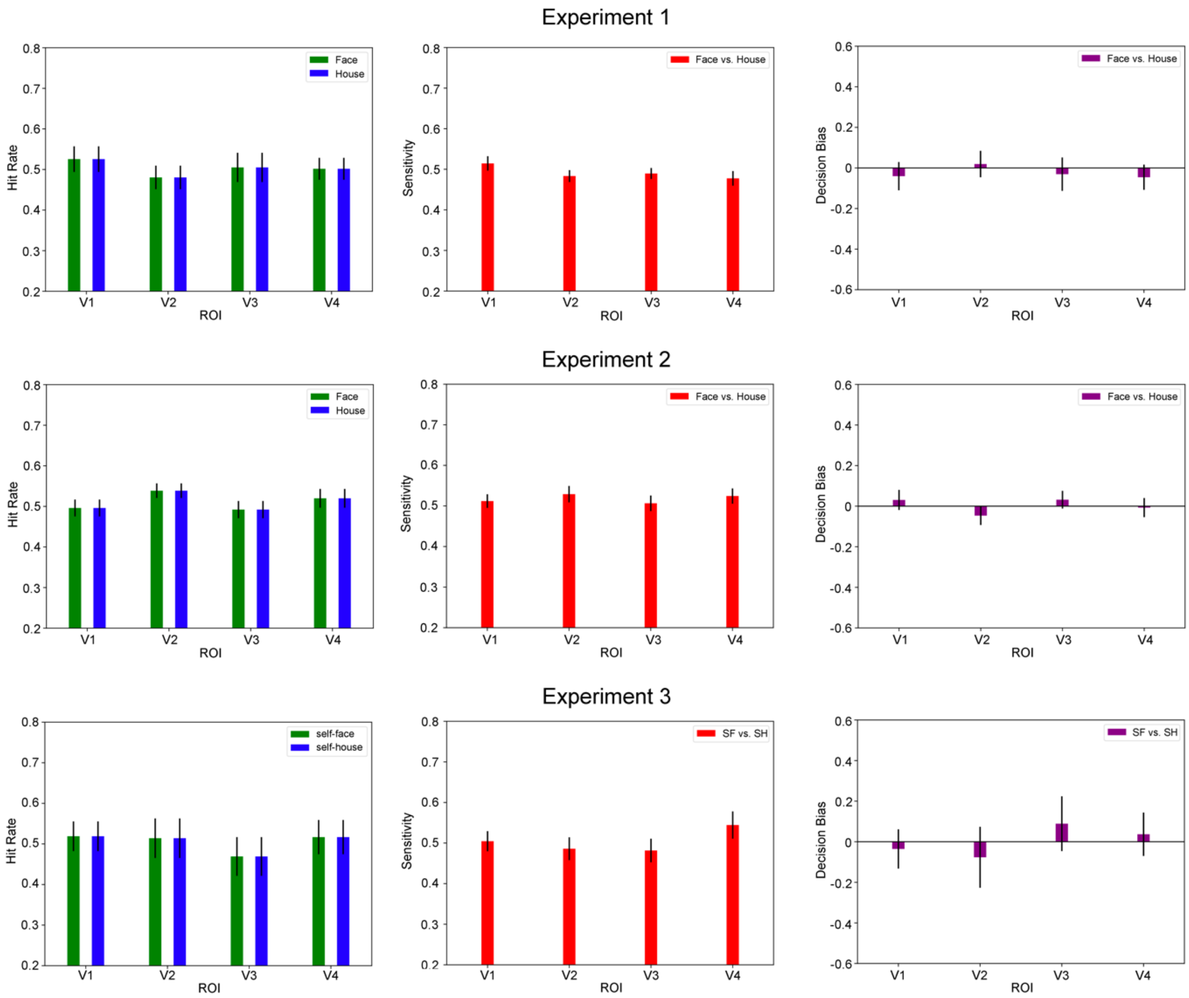

Supplementary Figure 3.

The results of the same analysis as in Supplementary Figure 2 for early visual areas (V1-V4).

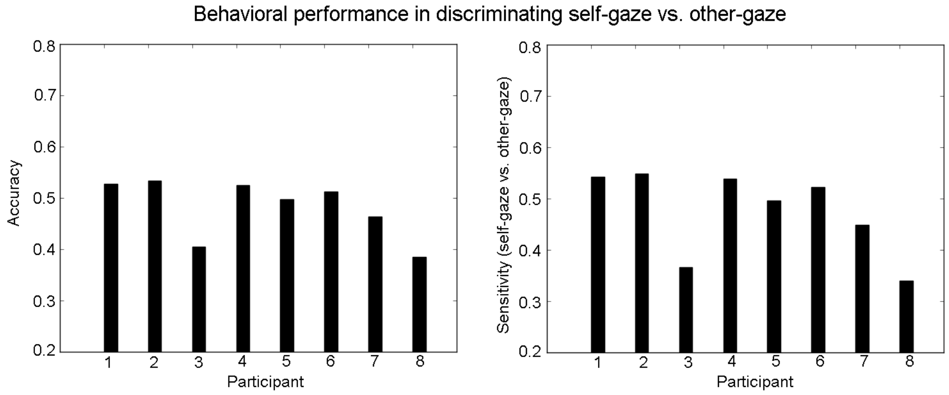

Supplementary Figure 4.

The behavioural accuracy (left) and the area under ROC curve (right) for each individual subject in discriminating Self-gaze versus Other-gaze.

Supplementary notes

Univariate analyses

Experiment 1 The repeated measures ANOVA with the factors ROI (FFA, PPA, FEF, SPL) and Fixation Pattern showed a significant main effect of ROI ($F(3,60)=154.30$, $p<.001$) - due to that the eye movement-defined FEF and SPL ROIs showed the strongest activation (ps < .001) - and an interaction between the two factors ($F(6,120)=3.33$, $p=.006$; Figure 3 A, left). The interaction consisted of stronger signal modulation caused by fixation pattern in PPA ($F(2,40)=13.20$, $p<.001$) than in the other areas (FFA: $F(2,40)=2.61$, $p=.086$, FEF: $F<1$, SPL: $F(2,40)=2.67$, $p=.082$). In the PPA, the strongest activation was observed for house fixation patterns (house vs. face: $p < .001$, house vs. inverted face, $p = .003$, face vs. inverted face: $p = .491$, paired-sample t test under Bonferroni-corrected $\alpha = .05$ for multiple comparisons).

The repeated measures ANOVA with the factors ROI (V1, V2, V3, V4) and Fixation Pattern (Face, House, inverted Face) showed a main effect of ROI, $F(3, 60) = 26.17$, $p < .001$, a main effect of Fixation Pattern, $F(2, 40) = 16.88$, $p < .001$, and an interaction between ROI and Fixation Pattern, $F(6, 120) = 5.30$, $p < .001$ (Figure 3 A, right). Post-hoc comparisons (paired-sample t test under Bonferroni-corrected $\alpha = .05$ for multiple comparisons) showed that the main effect of ROI was due to stronger signal change in V3 and V4 than in V1 and V2 (all $p < .001$), as well as stronger signal change in V2 than in V1 ($p = .040$), the main effect of Fixation Pattern was due to stronger activation for House than Face and inverted Face (both $p < .001$) relative to a null difference between Face and inverted Face ($p > .99$). Given the interaction, a separate ANOVA with the factor Fixation Pattern was conducted for each of the four ROIs, respectively. For V1, the main effect of Fixation Pattern was significant, $F(2, 40) = 18.70$, $p < .001$, which was due to stronger activation for House (Face vs. House: $p < .001$, inverted Face vs. House: $p < .001$, Face vs. inverted Face: $p = .876$). For V2, the main effect of Fixation Pattern was significant, $F(2, 40) = 15.60$, $p < .001$, which was due to stronger activation for House (Face vs. House: $p < .001$, inverted Face vs. House: $p < .001$, Face vs. inverted Face: $p = .904$). For V3, the main effect of Fixation Pattern was significant, $F(2, 40) = 15.70$, $p < .001$, which was due to stronger activation for House (Face vs. House: $p < .001$, inverted Face vs. House: $p < .001$, Face vs. inverted Face: $p > .99$). For V4, the main effect of Fixation Pattern was significant, $F(2, 40) = 11.00$, $p < .001$, which was due to stronger activation for House (Face vs. House: $p < .001$, inverted Face vs. House: $p < .001$, Face vs. inverted Face: $p > .99$, paired-sample t test under Bonferroni-corrected at $\alpha = .05$ for multiple comparisons in each area). These results suggested that the early visual areas were consistently biased for House Fixation Pattern, but this bias weakened along the visual pathway, as shown by the decreasing F value from V1 to V2/V3, and from V2/V3 to V4.

Experiment 2 The repeated measures ANOVA with the factors ROI (FFA, PPA, FEF, SPL) and Fixation Pattern (Face, House, inverted Face) revealed only a significant main effect of ROI, $F(3,60)=154.30$, $p<.001$ (Figure 3 B, left). As expected, activation was higher in the frontoparietal than the occipitotemporal ROIs. The post-hoc comparisons (paired-sample t test under Bonferroni-corrected $\alpha = .05$ for multiple comparisons) showed that the main effect of ROI was caused by stronger signal changes in SPL than the other three areas (all $p < .001$), and stronger signal changes in FEF than FFA and PPA (both $p < .001$). Neither the main effect of fixation pattern nor the interaction reached significance (both $F < 1$).

The repeated measures ANOVA with the factors ROI (V1, V2, V3, V4) and Fixation Pattern (Face, House, inverted Face) showed that only the main effect of ROI was significant, $F(3, 51) = 10.72$, $p < .001$, whereas neither the main effect of Fixation Pattern, $F(2, 34) = 1.07$, $p = .356$, nor the interaction, $F(6, 102) = 1.65$, $p = 0.141$ reached significance (Figure 3 B, right). Post-hoc comparisons (paired-sample t test under Bonferroni-corrected $\alpha = .05$ for multiple comparisons) showed that the main effect of ROI was due to stronger signal changes in V3 than in V1 ($p < .001$) and V2 ($p = .008$), stronger signal changes in V4 than V1 ($p < .001$), whereas none of the other pair-wise comparisons was significant, all $p > .086$.

Experiment 3 The repeated measures ANOVA with the factors ROI (V1, V2, V3, V4) and Fixation Pattern (Self-face, Self-house, Other-face, Other-house) showed a main effect of ROI, $F(3, 45) = 3.39$, $p = .026$ (Figure 3 C, right). Post-hoc comparisons (paired-sample t test under Bonferroni-corrected $\alpha = .05$ for multiple comparisons) showed that this main effect was caused by stronger signal change in V4 than V1 ($p = .052$) and V3 ($p = .055$). None of the other post-hoc comparisons reached significance, all $p > .924$. The main effect of Fixation Pattern, $F(3, 45) = 1.35$, $p =.269$ was not significant, whereas there was an interaction between the two factors, $F(9, 135) = 2.52$, $p = .011$. A further repeated measures ANOVA with only the factor Fixation Pattern did not real significant effect in V1 ($F < 1$), V2 ($F < 1$), or V4 ($F(3,45)= 1.90$, $p=.144$). There was only a trend of effect in V3, $F(3, 45) = 2.75$, $p=.054$. However, the post-hoc comparisons (paired-sample t test under Bonferroni-corrected $\alpha = .05$ for multiple comparisons) on the four fixation patterns did not reveal any significant effects (all $p > .08$).

Multivariate analyses

We ran whole-brain search light analyses for the classification of face vs. house in Experiment 1 and Experiment 2, and whole-brain search light analysis for the classification of self-face vs. self-house in Experiment 3. For all the search light analyses, the radius was set to 4 mm, and the permutation procedure was the same as in Stelzer et al., 2013 (1). No cluster was obtained from the whole-brain search light under the conventional threshold ($p < 0.001$ at voxel-level, $p < 0.05$ at cluster level with FWE correction, permutation testing) in any of the three experiments.

References

1. Stelzer, J., Chen, Y., & Turner, R. Statistical inference and multiple testing correction in classification-based multi-voxel pattern analysis (MVPA): random permutations and cluster size control. *Neuroimage* **65**, 69-82 (2013).
