# Supplementary material for: Individual face- and house-related eye movement patterns distinctively activate FFA and PPA
Source: Nat Commun. 2019 Dec 4;10:5532. doi: 10.1038/s41467-019-13541-3 (PMC6892816; doi:10.1038/s41467-019-13541-3)
Supplement: Supplementary file 3 — Reporting Summary [file 41467_2019_13541_MOESM3_ESM.pdf]

nature research

Corresponding author(s): Stefan Pollmann

Last updated by author(s): Nov 7, 2019

Reporting Summary

Nature Research wishes to improve the reproducibility of the work that we publish. This form provides structure for consistency and transparency in reporting. For further information on Nature Research policies, see Authors & Referees and the Editorial Policy Checklist.

Statistics

For all statistical analyses, confirm that the following items are present in the figure legend, table legend, main text, or Methods section.

n/a	Confirmed	
☐	☒	The exact sample size (*n*) for each experimental group/condition, given as a discrete number and unit of measurement
☐	☒	A statement on whether measurements were taken from distinct samples or whether the same sample was measured repeatedly
☐	☒	The statistical test(s) used AND whether they are one- or two-sided<br>*Only common tests should be described solely by name; describe more complex techniques in the Methods section.*
☐	☒	A description of all covariates tested
☐	☒	A description of any assumptions or corrections, such as tests of normality and adjustment for multiple comparisons
☐	☒	A full description of the statistical parameters including central tendency (e.g. means) or other basic estimates (e.g. regression coefficient) AND variation (e.g. standard deviation) or associated estimates of uncertainty (e.g. confidence intervals)
☐	☒	For null hypothesis testing, the test statistic (e.g. F, t, r) with confidence intervals, effect sizes, degrees of freedom and P value noted<br>*Give P values as exact values whenever suitable.*
☒	☐	For Bayesian analysis, information on the choice of priors and Markov chain Monte Carlo settings
☒	☐	For hierarchical and complex designs, identification of the appropriate level for tests and full reporting of outcomes
☐	☒	Estimates of effect sizes (e.g. Cohen's d, Pearson's r), indicating how they were calculated

Our web collection on statistics for biologists contains articles on many of the points above.

Software and code

Policy information about availability of computer code

Data collection	The task was programmed with PsychoPy (https://www.psychopy.org/).
Data analysis	Data analysis was performed with FSL5.0 (https://fsl.fmrib.ox.ac.uk/fsl/fslwiki) and PyMVPA (http://www.pymvpa.org/).

For manuscripts utilizing custom algorithms or software that are central to the research but not yet described in published literature, software must be made available to editors/reviewers. We strongly encourage code deposition in a community repository (e.g. GitHub). See the Nature Research guidelines for submitting code & software for further information.

Data

Policy information about availability of data

All manuscripts must include a data availability statement. This statement should provide the following information, where applicable:

- Accession codes, unique identifiers, or web links for publicly available datasets
- A list of figures that have associated raw data
- A description of any restrictions on data availability

All relevant data and analysis codes can be made available by the authors on request.

Field-specific reporting

Please select the one below that is the best fit for your research. If you are not sure, read the appropriate sections before making your selection.

☒ Life sciences ☐ Behavioural & social sciences ☐ Ecological, evolutionary & environmental sciences

For a reference copy of the document with all sections, see nature.com/documents/nr-reporting-summary-flat.pdf

Life sciences study design

All studies must disclose on these points even when the disclosure is negative.

Sample size	No statistical tests could be performed to predetermine the sample size, because no comparable study existed. Sample size was chosen in accordance with the standard range in the field.
Data exclusions	Established exclusion criteria were applied for excessive head motion and the poor recording of eye-tracking data in the scanner (see online methods).
Replication	The decoding results in FFA and PPA were reliably replicated in three experiments.
Randomization	Participants were recruited by MRI technicians who had no prior knowledge of the current study, and hence no randomization was performed for participants. Trial order and condition order were fully randomized for each subject.
Blinding	Data collection and analysis were not performed blindly to the conditions of the experiment. Blinding is not a conventional procedure for this kind of research.

Reporting for specific materials, systems and methods

We require information from authors about some types of materials, experimental systems and methods used in many studies. Here, indicate whether each material, system or method listed is relevant to your study. If you are not sure if a list item applies to your research, read the appropriate section before selecting a response.

Materials & experimental systems

n/a	Involved in the study	
☐	☐	Antibodies
☐	☐	Eukaryotic cell lines
☐	☐	Palaeontology
☐	☐	Animals and other organisms
☐	☒	Human research participants
☐	☐	Clinical data

Methods

n/a	Involved in the study	
☐	☐	ChIP-seq
☐	☐	Flow cytometry
☐	☒	MRI-based neuroimaging

Antibodies

Antibodies used	*Describe all antibodies used in the study; as applicable, provide supplier name, catalog number, clone name, and lot number.*
Validation	*Describe the validation of each primary antibody for the species and application, noting any validation statements on the manufacturer's website, relevant citations, antibody profiles in online databases, or data provided in the manuscript.*

Eukaryotic cell lines

Policy information about cell lines

Cell line source(s)	*State the source of each cell line used.*
Authentication	*Describe the authentication procedures for each cell line used OR declare that none of the cell lines used were authenticated.*
Mycoplasma contamination	*Confirm that all cell lines tested negative for mycoplasma contamination OR describe the results of the testing for mycoplasma contamination OR declare that the cell lines were not tested for mycoplasma contamination.*
Commonly misidentified lines (See ICLAC register)	*Name any commonly misidentified cell lines used in the study and provide a rationale for their use.*

Palaeontology

Specimen provenance	*Provide provenance information for specimens and describe permits that were obtained for the work (including the name of the issuing authority, the date of issue, and any identifying information).*
Specimen deposition	*Indicate where the specimens have been deposited to permit free access by other researchers.*

Dating methods

If new dates are provided, describe how they were obtained (e.g. collection, storage, sample pretreatment and measurement), where they were obtained (i.e. lab name), the calibration program and the protocol for quality assurance OR state that no new dates are provided.

☐ Tick this box to confirm that the raw and calibrated dates are available in the paper or in Supplementary Information.

Animals and other organisms

Policy information about studies involving animals; ARRIVE guidelines recommended for reporting animal research

Laboratory animals	*For laboratory animals, report species, strain, sex and age OR state that the study did not involve laboratory animals.*
Wild animals	*Provide details on animals observed in or captured in the field; report species, sex and age where possible. Describe how animals were caught and transported and what happened to captive animals after the study (if killed, explain why and describe method; if released, say where and when) OR state that the study did not involve wild animals.*
Field-collected samples	*For laboratory work with field-collected samples, describe all relevant parameters such as housing, maintenance, temperature, photoperiod and end-of-experiment protocol OR state that the study did not involve samples collected from the field.*
Ethics oversight	*Identify the organization(s) that approved or provided guidance on the study protocol, OR state that no ethical approval or guidance was required and explain why not.*

Note that full information on the approval of the study protocol must also be provided in the manuscript.

Human research participants

Policy information about studies involving human research participants

Population characteristics	Fifty-five healthy subjects (24 female, mean age: 26.3, SD = 3.8) with normal or corrected-to-normal vision and normal color vision.
Recruitment	Participants were recruited by MRI technicians who had no prior knowledge of the current study.
Ethics oversight	The ethics committee of the University of Magdeburg

Note that full information on the approval of the study protocol must also be provided in the manuscript.

Clinical data

Policy information about clinical studies

All manuscripts should comply with the ICMJE guidelines for publication of clinical research and a completed CONSORT checklist must be included with all submissions.

Clinical trial registration	*Provide the trial registration number from ClinicalTrials.gov or an equivalent agency.*
Study protocol	*Note where the full trial protocol can be accessed OR if not available, explain why.*
Data collection	*Describe the settings and locales of data collection, noting the time periods of recruitment and data collection.*
Outcomes	*Describe how you pre-defined primary and secondary outcome measures and how you assessed these measures.*

ChIP-seq

Data deposition

☐ Confirm that both raw and final processed data have been deposited in a public database such as GEO.

☐ Confirm that you have deposited or provided access to graph files (e.g. BED files) for the called peaks.

Data access links<br>*May remain private before publication.*	*For "Initial submission" or "Revised version" documents, provide reviewer access links. For your "Final submission" document, provide a link to the deposited data.*
Files in database submission	*Provide a list of all files available in the database submission.*
Genome browser session<br>(e.g. UCSC)	*Provide a link to an anonymized genome browser session for "Initial submission" and "Revised version" documents only, to enable peer review. Write "no longer applicable" for "Final submission" documents.*

Methodology

Replicates	*Describe the experimental replicates, specifying number, type and replicate agreement.*

Sequencing depth	*Describe the sequencing depth for each experiment, providing the total number of reads, uniquely mapped reads, length of reads and whether they were paired- or single-end.*
Antibodies	*Describe the antibodies used for the ChIP-seq experiments; as applicable, provide supplier name, catalog number, clone name, and lot number.*
Peak calling parameters	*Specify the command line program and parameters used for read mapping and peak calling, including the ChIP, control and index files used.*
Data quality	*Describe the methods used to ensure data quality in full detail, including how many peaks are at FDR 5% and above 5-fold enrichment.*
Software	*Describe the software used to collect and analyze the ChIP-seq data. For custom code that has been deposited into a community repository, provide accession details.*

Flow Cytometry

Plots

Confirm that:

- [ ] The axis labels state the marker and fluorochrome used (e.g. CD4-FITC).
- [ ] The axis scales are clearly visible. Include numbers along axes only for bottom left plot of group (a 'group' is an analysis of identical markers).
- [ ] All plots are contour plots with outliers or pseudocolor plots.
- [ ] A numerical value for number of cells or percentage (with statistics) is provided.

Methodology

Sample preparation	*Describe the sample preparation, detailing the biological source of the cells and any tissue processing steps used.*
Instrument	*Identify the instrument used for data collection, specifying make and model number.*
Software	*Describe the software used to collect and analyze the flow cytometry data. For custom code that has been deposited into a community repository, provide accession details.*
Cell population abundance	*Describe the abundance of the relevant cell populations within post-sort fractions, providing details on the purity of the samples and how it was determined.*
Gating strategy	*Describe the gating strategy used for all relevant experiments, specifying the preliminary FSC/SSC gates of the starting cell population, indicating where boundaries between "positive" and "negative" staining cell populations are defined.*

- [ ] Tick this box to confirm that a figure exemplifying the gating strategy is provided in the Supplementary Information.

Magnetic resonance imaging

Experimental design

Design type	Event-related. Trial order was fully randomized.
Design specifications	In Experiments 1 and 2, there was only one block with 168 trials (56 trials per condition) and the block lasted for 26 min. In Experiment 3, there were 14 blocks with 28 trials (7 trials per condition) and each block lasted for 3.8 min.
Behavioral performance measures	In each experiment, the correct response in detecting the small black dot was recorded. The proportion of the correct responses (in percentage) were reported as the effect sizes of behavioral performance.

Acquisition

Imaging type(s)	functional and structural
Field strength	3T
Sequence & imaging parameters	In Experiments 1 and 2, MR-data were acquired at a 3T Siemens Trio MR-scanner equipped with an 8-channel head-coil. For anatomical coregistration, a T1 weighted image was recorded before the functional imaging (192 sagittal slices, 256 x 256 1mm isotropic voxels, TR=2500ms, TE=4.77, FA=7°). The same echo-planar imaging sequence was used for the main experiment as well as for the localizer (34 transversal slices, 3.5 mm isotropic voxels, matrix size = 64 voxels, TR = 2000 ms, TE = 30 ms, flip angle = 80°, interleaved slice acquisition).<br>In Experiment 3, MR-data were acquired at a 3T Philips Achieva dStream MR-scanner equipped with a 32-channel head-coil. For anatomical coregistration, a T1 weighted image was recorded before the functional imaging (192 sagittal slices, 256 x 256 1mm isotropic voxels, TR=2300ms, TE=4.65 ms, FA=8°). The same echo-planar imaging sequence was used for

the main experiment as well as for the localizer (35 transversal slices, 3 mm isotropic voxels, matrix size = 80 voxels, TR = 2000 ms, TE = 30 ms, flip angle = 90°, ascending slice acquisition).

Area of acquisition: Slices were aligned parallel to the anterior and posterior commissures and covered the whole neocortex.

Diffusion MRI: ☐ Used ☒ Not used

Preprocessing

Preprocessing software: FSL5.0

Normalization: The MVPA was conducted on individually defined ROIs (FFA, PPA, FEF, SPL) and did not involve normalization.
For the MVPA on early visual areas and the univariate analysis, each participant's functional images were aligned to the individual structural high-resolution image using a 12 degrees-of-freedom (DOF) affine transformation, and were coregistered to the MNI152 standard template (2mm isometric voxel resolution) by applying a 12 DOF affine transformation.

Normalization template: MNI152

Noise and artifact removal: High-pass filtering, motion correction, modelling head movement parameters as nuisance regressors.

Volume censoring: No volume censoring performed.

Statistical modeling & inference

Model type and settings: Single-subject level: multivariate GLM
Group-level: permutation-based significance testing

Effect(s) tested: We tested if the different gaze patterns can be discriminated by activity patterns in pre-defined ROIs (e.g., FFA and PPA). This hypothesis was tested by examining if the prediction accuracy exceeded chance-level that derived from permutations. We also tested if the individual prediction accuracies correlated with the eye-movement patterns by performing Pearson correlation.

Specify type of analysis: ☐ Whole brain ☒ ROI-based ☐ Both

Anatomical location(s): FFA and PPA were obtained by a visual localizer task; FEF, SPL were based on both the task main effect and meta-analysis (http://neurosynth.org/). Early visual areas (V1-V4) were based on probabilistic maps (Eickhoff et al., 2007).

Statistic type for inference (See Eklund et al. 2016): voxel-wise inference

Correction: permutation, Bonferroni correction

Models & analysis

n/a	Involved in the study
☐	☐ Functional and/or effective connectivity
☐	☐ Graph analysis
☐	☒ Multivariate modeling or predictive analysis

Functional and/or effective connectivity: Not applicable.

Graph analysis: Not applicable.

Multivariate modeling and predictive analysis: Independent variable: face- and house-associated gaze patterns.
Feature selection: independently obtained ROI. Voxels in early visual areas were thresholded based on the probability value.
Model: multivariate GLM.
Evaluation: data samples were divided into independent training set and test set. Following the recommendation of Jamalabadi et al. (2016), twofold cross-validation was performed to generate the prediction accuracy.
